# The ubiquitin proteasomal system: a potential target for the management of Alzheimer's disease

**DOI:** 10.1111/jcmm.12817

**Published:** 2016-03-29

**Authors:** Kundlik Gadhave, Nityanand Bolshette, Ashutosh Ahire, Rohit Pardeshi, Krishan Thakur, Cristiana Trandafir, Alexandru Istrate, Sahabuddin Ahmed, Mangala Lahkar, Dafin F. Muresanu, Maria Balea

**Affiliations:** ^1^ Laboratory of NeurobiologyDepartment of Pharmacology and ToxicologyNational Institute of Pharmaceutical Education and Research (NIPER)Gauhati Medical CollegeGuwahatiAssamIndia; ^2^ Institutional Level Biotech hub (IBT hub)Department of BiotechnologyNational Institute of Pharmaceutical Education and Research (NIPER)Gauhati Medical CollegeGuwahatiAssamIndia; ^3^ Faculty of MedicineUniversity of Medicine and Pharmacy “Iuliu Hatieganu”Cluj‐NapocaRomania; ^4^ Department of Clinical NeurosciencesUniversity of Medicine and Pharmacy “Iuliu Hatieganu”Cluj‐NapocaRomania; ^5^ “RoNeuro” Institute for Neurological Research and DiagnosticCluj‐NapocaRomania

**Keywords:** ubiquitin, Alzheimer's disease, amyloid β, chaperones

## Abstract

The cellular quality control system degrades abnormal or misfolded proteins and consists of three different mechanisms: the ubiquitin proteasomal system (UPS), autophagy and molecular chaperones. Any disturbance in this system causes proteins to accumulate, resulting in neurodegenerative diseases such as amyotrophic lateral sclerosis, Alzheimer's disease (AD), Parkinson's disease, Huntington's disease and prion or polyglutamine diseases. Alzheimer's disease is currently one of the most common age‐related neurodegenerative diseases. However, its exact cause and pathogenesis are unknown. Currently approved medications for AD provide symptomatic relief; however, they fail to influence disease progression. Moreover, the components of the cellular quality control system represent an important focus for the development of targeted and potent therapies for managing AD. This review aims to evaluate whether existing evidence supports the hypothesis that UPS impairment causes the early pathogenesis of neurodegenerative disorders. The first part presents basic information about the UPS and its molecular components. The next part explains how the UPS is involved in neurodegenerative disorders. Finally, we emphasize how the UPS influences the management of AD. This review may help in the design of future UPS‐related therapies for AD.

## Introduction

Biosynthesized proteins often misfold because of destabilizing mutations, stress or metabolic changes 1 and cannot perform their biological functions. Protein misfolding is prevented by the cellular quality control system, which consists of the ubiquitin proteasomal pathway (26S pathway or UPP), autophagy and molecular chaperones 2, 3. These mechanisms, which are often referred to as the cellular proteostasis network, prevent the accumulation of toxic levels of unfolded or misfolded proteins 4 by breaking the proteins down into polypeptide chains 5, 6. Any disturbance in this system causes proteins in different parts of the body to accumulate and promotes severe neurodegenerative disorders, such as Alzheimer's disease (AD), Parkinson's disease (PD) and prion or polyglutamine diseases 2, 7.

Presently, AD is one of the best described age‐related neurodegenerative diseases. However, its potential cause is not fully understood. Alzheimer's disease is characterized by a progressive loss of memory and object recognition, dementia and changes in behavior and speech. It is associated with the synthesis of defective proteins, errors in their transport to membranes and oxidative damage. Researchers have suggested that the aggregation of amyloid beta (Aβ) initiates this disease. Amyloid beta is formed when amyloid precursor protein (APP) 4, 6 undergoes successive enzymatic cleavages and is transported into the extracellular space. Overproduction or defective clearance of Aβ causes it to progressively accumulate. Amyloid beta self‐aggregates into oligomers, which obstruct proteasome function and disturb neuronal processes. Furthermore, Aβ inhibits the proteasomal pathway and promotes the formation and accumulation of hyperphosphorylated tau, a highly soluble microtubule‐binding protein 8. Normally, tau stabilizes the microtubule network within axons and facilitates the axonal transport of organelles, neurotransmitters and other cellular constituents that act as trophic factors 9, 10 Hyperphosphorylated tau self‐aggregates into neurofibrillary tangles (NFTs) and impairs axonal transport and neuronal function 8.

Protein degradation is primarily performed by proteasomes in the cytosolic and nuclear compartments 11. Changes in UPP‐based protein degradation lead to pleiotropic effects, such as neurodegeneration, synaptic dysfunction and neuron death 12. Recently, many studies have suggested that UPP affects the pathogenesis of AD 12, 13. First, ubiquitinated proteins are present in AD. In addition, misfolded proteins within all tissues are normally degraded by the UPP 8, 9. However, the exact underlying mechanism of this process continues to be investigated.

## The ubiquitin proteasomal system

Intracellular protein degradation is a selective process 14 that, in eukaryotic cells, is catalyzed by proteasome, the major component of the ubiquitin proteasomal system (UPS) 8, 15. Ubiquitin proteasomal system‐mediated degradation is the primary proteolytic mechanism for normal and abnormal synaptic proteins. The UPS includes five steps: activation, conjugation, recognition, ubiquitin removal by specific deubiquitinating enzymes (DUBs) and substrate degradation by the proteasome 16, 17.

The UPS controls protein degradation by proteasome‐mediated proteolysis and regulates protein function *via* multiple types of ubiquitination. The UPS degrades more than 80% of normal and abnormal intracellular proteins 18. Within tissues, most intracellular proteins are degraded by the UPP, whereas extracellular proteins and several proteins on the cell surface are endocytosed and degraded *via* lysosomes 19. The UPP controls many cellular processes: the cell cycle, DNA transcription and repair, apoptosis and quality control 20, 21. It also maintains proteostasis during aging and disease and prevents protein misfolding and aggregation 22.

### Ubiquitination and deubiquitination

**Figure 1:**
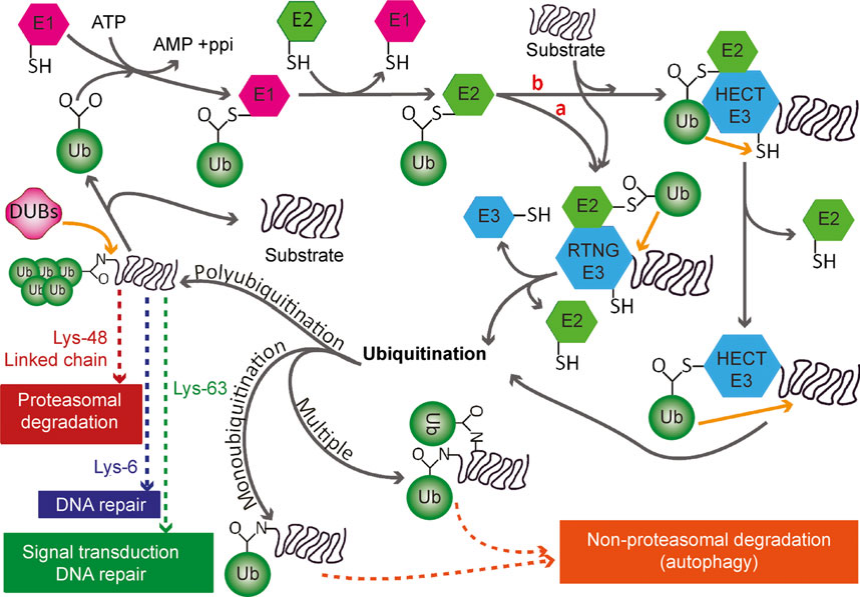
General outline of ubiquitination. Ubiquitination is the covalent attachment of the glycine residue of ubiquitin to the lysine residue of the target protein *via* an isopeptide bond. This process is mediated by E1, E2 and E3 enzymes. E1 ligases forms thioester bonds with ubiquitin, which is subsequently transferred to E2 ligases through a thioester linkage. E3 ligases are grouped into two types: HECT type and RING‐finger/adaptor type. Only the HECT domain E3 ligase forms a covalent bond with ubiquitin during polyubiquitination of abnormal proteins, whereas the RING‐finger E3 ligases directly transfer ubiquitin from the associated E2 enzyme to the substrate protein. Proteins can be mono‐, multiple‐ or poly‐ubiquitinated. Mono‐ and multiple‐ubiquitinated proteins are degraded by lysosomes (autophagy), whereas the degradation of polyubiquitinated proteins depends on the type of ubiquitin linkage (K48, K63, K6, K11, K27, K29 and K33). K48‐linked polyubiquitinated proteins are generally degraded by the proteasomal pathway. Polyubiquitination *via* K6 is associated with DNA repair; *via* K11, with endoplasmic reticulum‐associated protein degradation and cell cycle regulation; *via* K27, with ubiquitin fusion degradation; *via* K29, with lysosomal degradation; and *via* K33, with kinase modification. DUBs remove ubiquitin from the substrate protein, and the monomers are recycled.

**Figure 2:**
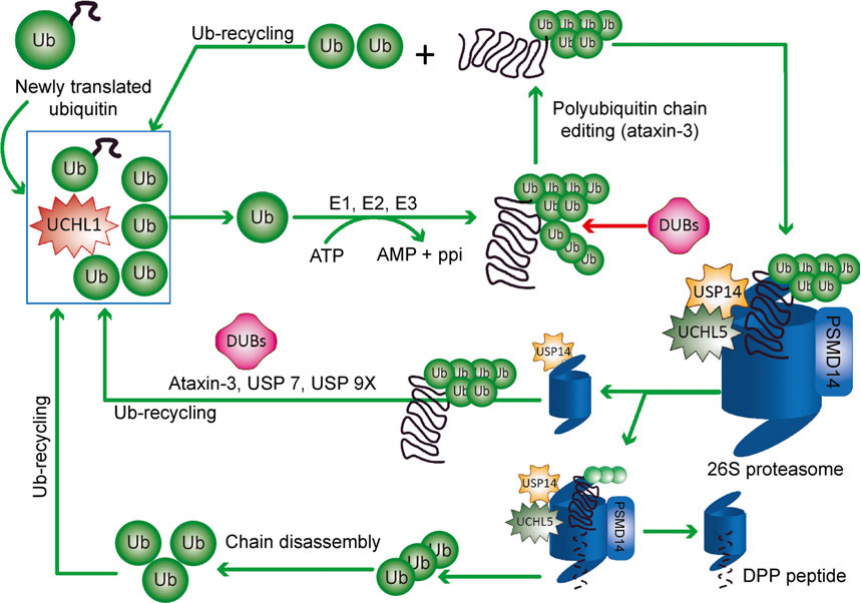
The deubiquitination process. Different types of deubiquitinase enzymes (DUBs) mediate the degradation of misfolded proteins. Newly translated ubiquitin (Ub) that is fused with the C‐terminal end of amino acids is cleaved by DUBs, such as UCHL1. UCHL1 maintains the pool of mono‐Ub and supplies ubiquitin for misfolded protein ubiquitination. Ubiquitin enzymes (E1, E2, and E3 enzymes) form the polyubiquitin chain on substrate proteins by adding mono‐UB. The polyubiquitinated chain is modified by DUBs, such as ataxin‐3, to ensure correct recognition of the substrate proteins by the 26S proteasome. The mono‐Ubs are again recycled back into the pool of mono‐Ub. DUBs, including USP14, work in two ways: (*i*) USP14 rescues polyubiquitinated proteins from degradation by the 26S proteasome, whereas DUBs such as USP7, ataxin‐3 and USP9X facilitate the deubiquitination of poly‐Ub chains and the recycling of mono‐Ubs back into the pool of mono‐Ub; and (*ii*) USP14 may also work in combination with other DUBs including PSMD14 and UCHL5, on the 26S proteasome to degrade misfolded proteins into small peptides. The poly‐Ub chain is disassembled to mono‐Ubs that are recycled back into the pool of mono‐Ub.

Ubiquitin is a 76‐amino‐acid protein with a molecular weight of 8.5 kD that is present in all tissues in a free or covalently conjugated form. Ubiquitination is a reversible process 23 that consists of the covalent attachment of the glycine residue of ubiquitin to a lysine residue of the target protein *via* an isopeptide bond through the actions of ubiquitin activating (E1), conjugating (E2) and ligating (E3) enzymes 2, 24. Recently, the activity of an E4 enzyme was described. E1 and E2 enzymes prepare ubiquitin for conjugation. E3 enzymes recognize the specific substrate and catalyze the transfer of activated ubiquitin to the substrate 19, 25. E4 enzymes catalyze the conjugation of additional ubiquitin monomers to form a polyubiquitin chain, usually through lysine 48 (K48) linkages 12 (Fig. 1). For 26S proteasome degradation, a polyubiquitin chain is made of four or more ubiquitin proteins that target a single substrate 8. There are two types of E3 ligases: HECT (homologous to E6‐associated protein C‐terminus) and RING‐finger/adaptor. Only the HECT domain E3 ligase forms a covalent bond with ubiquitin through a thioester intermediate during polyubiquitination (the transfer of ubiquitin to the substrate) of abnormal proteins. The RING‐finger E3 ligase directly transfers ubiquitin from its associated E2 enzyme to the substrate 16. The fate of ubiquitinated proteins depends on the type of linkage with ubiquitin (K48, K63, K6, K11, K27, K29 and K33). K48‐linked polyubiquitinated proteins are generally degraded through the proteasomal pathway, whereas K63‐linked polyubiquitinated proteins are degraded *via* the lysosomal pathway 26. The different types of ubiquitin linkage exhibit the following associations: K6 with DNA repair, K11 with endoplasmic reticulum (ER)‐associated protein degradation and cell cycle regulation, K27 with ubiquitin fusion and degradation, K29 with lysosomal degradation and K33 with kinase modification 22. After the proteasome recognizes the polyubiquitinated substrate, DUBs deubiquitinate the polyubiquitin chain at different levels of the UPP. Deubiquitination may occur before or after DUBs recognize the substrate on the 26S proteasome 14. After they recognize the substrate, the catalytic core degrades the substrate, and the DUBs recycle ubiquitin and preserve monoubiquitin for additional ubiquitination 14. Deubiquitination is necessary when newly translated ubiquitin binds with the C‐terminal end of amino acids or is cleaved by ubiquitin C‐terminal hydrolase 1 (UCHL1). Approximately, 100 DUBs are found in eukaryotes 27. Of them, 27 are in the nervous system, and seven are in the proteasomal system 14 (Fig. 2). Deubiquitinating enzymes are grouped into five different classes: (*i*) ubiquitin C‐terminal hydrolases (UCHs), *e.g*. UCHL1, UCHL3, and UCHL5/UCH37; (*ii*) ubiquitin‐specific proteases (USPs), *e.g*. USP7, USP9x, and USP14; (*iii*) Machado‐Joseph disease protease, *e.g*. ataxin‐3; (*iv*) otubain proteases (OTUs), *e.g*. otubain 1 and otubain 2 and (*v*) metallo‐enzymes (JAMMs), *e.g*. PSMD14/Rpn11 and JAB1/MPN/Mov34 14, 28.

Ubiquitin chain trimming promotes or inhibits proteasomal degradation. The enzymes involved in this process (especially DUBs) are potential therapeutic targets for the regulation of proteasomal degradation and the treatment of AD 14, 27.

### The 26S proteasome

The proteasome is a multisubunit protein complex that includes parts of the cytoplasm and the nucleus. There are several thousands of proteasomes, which are responsible for intracellular protein degradation 29 of normal and misfolded cytoplasmic proteins 30. Within the cytoplasm, the proteasome is associated with the centrosome, the cytoskeletal network and the outer surface of the ER, whereas in the nucleus, it is present throughout the nucleoplasm 31. Two types of proteasomes exist in eukaryotes: a smaller 20S proteasome that degrades unfolded proteins and a larger 26S proteasome that degrades folded proteins in an ATP‐dependent or ‐independent manner 30, 32.

A modification of the 26S proteasome, the immunoproteasome, is also involved in the pathogenesis of AD *via* neuroinflammation 33, 34. The 26S proteasome is a 2.5‐MDa multicatalytic protease complex that recognizes and degrades ubiquitin‐tagged substrates 19. It consists of a 20S core particle (CP) (700 kD) that is associated with one or two 19S regulatory particles (RPs) 18, 20. After the substrates are ubiquitinated, the 26S proteasome recognizes them in the presence of the lid subunits of the 19S RP, Rpn10/S5a and Rpn13/Adrm1 27. Then, the ubiquitinated substrates are deubiquitinated by Rpn11 in the presence of the DUBs USP14, UCHL5, PSMD14, UCHL1, Ataxin‐3, USP7 and USP9x 8, 14. The proteins are then unfolded through ATP hydrolysis in the 19S RP and translocated into the 20S CP for degradation 29.

**Figure 3:**
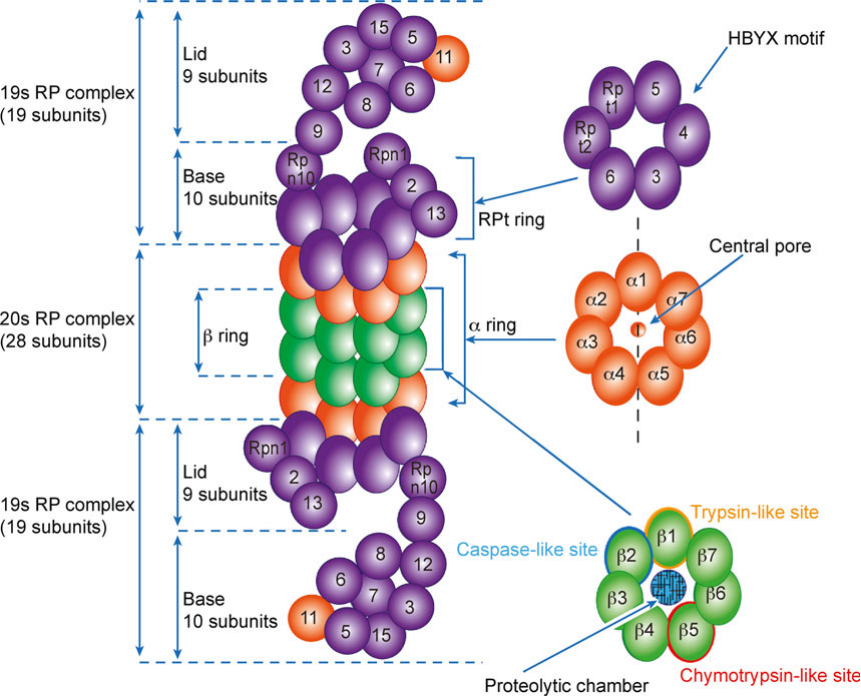
Basic molecular construction of the 26S proteasome. The proteasome consists of the 19S regulatory particle (19S‐RP) and the 20S core particle (20S‐CP). The 19S‐RP consists of 19 subunits that are divided into lid and base and is responsible for specific polyubiquitinated substrate recognition, deubiquitination, unfolding, and translocation to a proteolytic chamber. The lid consists of nine subunits (Rpn3, 5‐9, 11, 12 and 15), whereas the base consists of 10 subunits (Rpt1‐6, Rpn10, and Rpn1, 2 and 13). Rpn10/S5a and Rpn13 (hRpn1, ADRM1 or ARM1) act as ubiquitin receptors and are important for ubiquitinated protein degradation. The Rpt ring (Rpt1‐6) is associated with three HbYX motifs. In the presence of HbYX motifs, RP associates with the α‐ring of the 20S‐CP and open its N‐terminal gate to enable substrate entry. The 20S‐CP complex consists of two outer α‐rings and two inner β‐rings that together include 28 subunits. The two outer α‐rings (α1‐α7) contain a total of 14 subunits that create a central pore/gate to enable the entry of unfolded proteins into the proteolytic chamber. The two inner β‐rings (β1‐β7) also contain a total of 14 subunits and form the proteolytic chamber where protein degradation occurs. Each β‐ring contains six proteolytic sites, two β1 (trypsin‐like site), two β2 (caspase‐like site) and two β5 (chymotrypsin‐like site); these sites are responsible for the degradation of the substrate protein.

The 19S RP is composed of two subcomplexes (lid and base), both of which contain at least 19 individual subunits. These subunits are responsible for specific substrate recognition, deubiquitination, and unfolding and translocation of the substrate into the proteolytic chamber for degradation 29. The lid consists of nine non‐ATPase subunits (Rpn3, 5‐9, 11, 12, and 15), and the base consists of six ATPase (Rpt1‐6) and four non‐ATPase (Rpn1, 2, 13, and 10) subunits 20 (Fig. 3). The primary function of the lid is to deubiquitinate the substrate: Rpn11 in the lid displays deubiquitinating activity 20 and aids ubiquitin recycling 24. The base subcomplex is composed of six homologous ATPase subunits (Rpt1‐6) and 4 non‐ATPase subunits (Rpn1, 2, 10 and 13). The base recognizes ubiquitin, binds to the substrate, promotes its unfolding and opens the channel of the 20S CP 35. The Rpn10/S5a and Rpn13/Adrm1 subunits of the base act as two integral receptors and coordinate binding of the proteasome to polyubiquitin chains 27. Rpn10 also stabilizes the bond between the lid and the base 20. Rpn2 is the largest subunit of the proteasome and stabilizes the 19S complex 27.

In several cases, the 19S RP can be replaced by the PA28/11S regulatory complex, which contains a single ring of seven subunits 36. Replacing three catalytic subunits in a standard proteasome forms an immunoproteasome 37. The immunoproteasome utilizes the 11S RP instead of the 19S RP to facilitate 20S gate opening and to stimulate substrate unfolding and translocation. In addition, the immunoproteasome has increased chymotrypsin‐like and trypsin‐like activities because of the six different ATPases (Rpt1‐6) at the base of the 19S RP that interact with the 20S catalytic chamber 38.

The 20S CP, also known as the 20S ‘core’ proteasome complex or 20S proteasome 39, contains peptidases that function through a threonine active site 40. Unlike the 26S proteasome, the 20S CP actively recognizes and degrades substrate proteins and is not reversibly inactivated by mild oxidative stress 41. In addition, the 20S CP can only degrade proteins that are already unfolded, whereas the 26S proteasome can degrade folded and functional proteins 42. The 20S CP is a barrel‐like structure and consists of four stacked heptameric rings (α, β, β, and α) 29. The outer two α‐rings consist of seven homologous subunits (α1‐α7), and the inner two β‐rings consist of seven homologous subunits (β1‐β7) 29. The β‐ring contains a wide pore at its center in which proteolysis occurs, and the α‐ring contains a narrow pore that allows the translocation of only unfolded proteins into the 20S CP 29. The two β‐rings in the 20S CP perform six proteolytic activities, two caspase‐like, two trypsin‐like and two chymotrypsin‐like, which are performed by the β1, β2 and β5 subunits respectively 43. A docking study 29 demonstrated that the binding of the 19S RP to the 20S CP occurs through an L‐shaped link between the base of the 19S RP and the α subunit of the 20S CP. This link forms between the putative ATPase densities and the side of the α2 subunit of the 20S CP 9, 44. Three additional links form between the 19S RP and the 20S CP. They are parallel to the long axis of the proteasome and extend from the putative ATPase region of the 19S RP to A5, A6 and the interface between A7 and A1 of the 20S CP. When the 19S RP binds to the 20S CP, the gate to the proteolytic chamber is opened through the displacement of the subunit within the α‐ring of the 20S CP 29, 45. The 20S core complex performs various catalytic activities, including cleaving the carboxy‐terminal side of hydrophobic, basic or acidic residues 46. 20S CP is activated by the ATPases and archaeal proteasome‐activating nucleotide (PAN), which consists of peptides with a C‐terminal hydrophobic tyrosine‐x (HbYX) motif 24. The N‐terminal tails of the α‐subunits of the 20S CP form a gate that opens when the HbYX peptide of the ATPase binds to the pockets of the 20S α‐ring 47. The C‐terminal peptides of the ATPases Rpt2 and Rpt5 induce and regulate gate opening 24, 48. The regulatory peptides of the PAN C‐terminus bind to the inner subunit pockets and open the gate of the 20S CP so that the substrate can enter the proteolytic chamber 24.

The 26S proteasome binds polyubiquitinated misfolded proteins, deubiquitinates them, unfolds misfolded proteins, opens the α‐gate of the 20S CP and subsequently degrades the misfolded proteins 35. Ubiquitin affects substrate susceptibility to proteasomal degradation in two ways: the ubiquitination level of the substrate promotes proteasome‐substrate interactions, and the ubiquitin chain structure influences substrate translocation to the 20S CP 49.

### The ubiquitin proteasomal pathway in neurodegenerative disorders

Neurodegenerative diseases are characterized by abnormal deposition of insoluble protein aggregates or inclusion bodies within neurons 50. Many of them are caused by the accumulation of abnormal, misfolded or aberrant proteins 51; protofibril formation 52; UPS dysfunction 53; excitotoxic insult 54, 55; oxidative and nitrosative stress 56; mitochondrial injury 57; synaptic failure 58 or altered metal homeostasis 59. The UPP controls many of these processes in the nervous system. Several of its components are linked to synaptic dysfunction and genetic mutations that lead to neuronal disorders 50: UPS impairment has been associated with synaptic dysfunction in AD, PD, Huntington's disease (HD), schizophrenia, amyotrophic lateral sclerosis, ataxia, Angelman syndrome, Wallerian degeneration and gracile axonal dystrophy 50, 60. Ubiquitinated protein aggregates cause UPS dysfunction or structural changes in protein substrates that prevent their recognition and degradation. This loss of degradation has pleiotropic effects on neurons, such as cell death, degeneration and synaptic malfunction 50. Synaptic plasticity impairment and its connection to the UPP have been more extensively studied in AD than in PD and HD 60.

**Figure 4:**
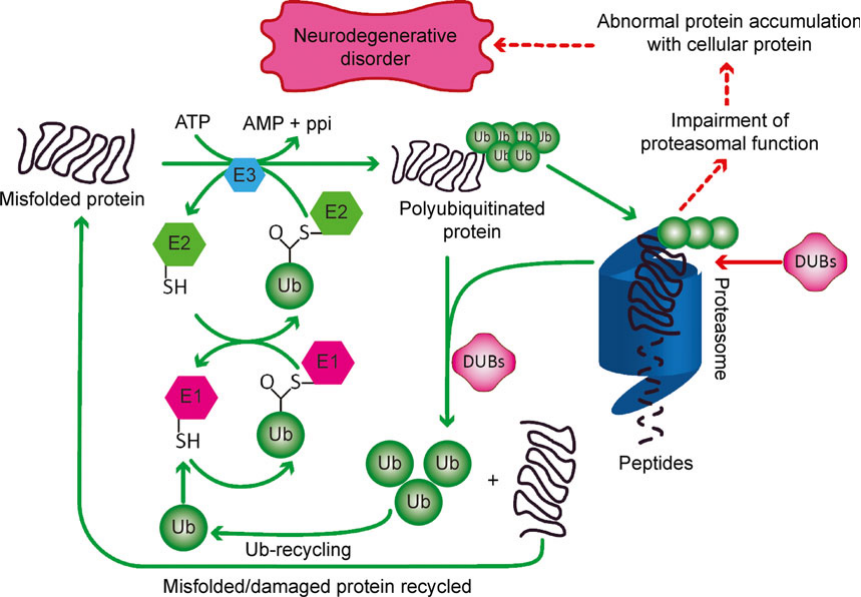
Delineation of the ubiquitin proteasome pathway in the pathogenesis of Alzheimer's disease. Improperly folded proteins are initially polyubiquitinated by the ubiquitin (Ub) enzymes E1 (Ub activating), E2 (Ub ligating) and E3 (Ub conjugating). These polyubiquitinated substrates are easily recognized by the 19S regulatory particle of the 26S proteasome. The polyubiquitinated protein is deubiquitinated by deubiquitinase enzymes (DUBs) and degraded into small peptides through 20S core particle. The polyubiquitinated proteins are deubiquitinated before they are recognized by the 26S proteasome. After they are recognized, they are polyubiquitinated again by the same mechanism and degraded by the 26S proteasome. DUBs convert poly‐Ub chains to Ub monomers, which are then recycled. Proteasome impairment decreases the degradation of amyloid beta (Aβ) and tau and promotes their oligomerization. The oligomers accumulate in neurons, resulting in neuronal death and Alzheimer's disease.

Alzheimer's disease is a progressive neurodegenerative disorder that is also the most frequent cause of dementia. It is characterized by the intracellular accumulation of Aβ oligomers and hyperphosphorylated tau and by the extracellular accumulation of high‐molecular‐weight deposits of Aβ peptides in fibrillar forms. These accumulations results in memory loss and severe cognitive decline 22, 50. *Via* the 26S proteasome, the UPP regulates Aβ metabolism and tau degradation 22. Its impairment in the neurons of AD patients causes ubiquitinated proteins to accumulate and the combinations of proteasome subunits to change 22, 60, which decreases proteasomal activity, decreases α‐secretase activity and produces Aβ 22 (Fig. 4).

Alzheimer's disease is characterized by progressive degeneration and loss of cortical and limbic neurons. Amyloid beta peptides form senile plaques in the extracellular space, and NFTs are present intracellularly 61. Proteasomal activity is decreased in the parahippocampal gyrus, the superior and middle temporal gyri and the inferior parietal lobe, suggesting that the UPP may be involved in the pathogenesis of AD 62.

## Treatment strategies for Alzheimer's disease

**Figure 5:**
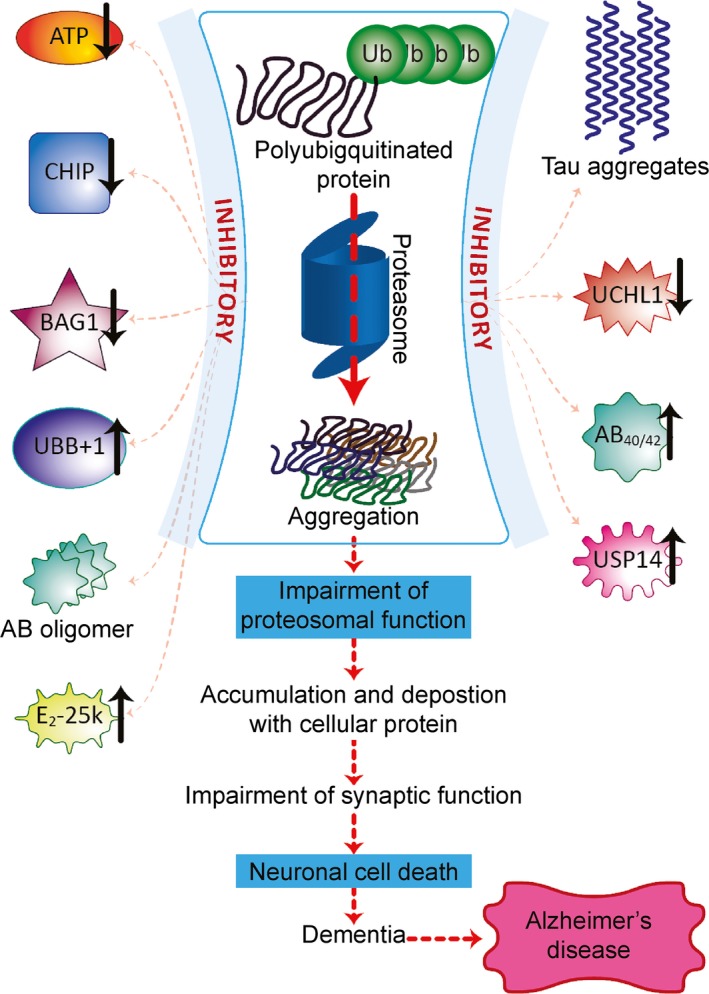
Approaches for the management of Alzheimer's disease. Inhibitors of the 26S proteasome cause Aβ and tau aggregation, which impairs proteasomal function and promotes the accumulation of Aβ and tau in neurons, resulting in neuronal death and Alzheimer's disease. ATP hydrolysis is necessary for both ubiquitination and 26S proteasomal degradation of ubiquitinated proteins. UBB ^+1^ is selectively expressed in neurons and competes with normal ubiquitin for binding to target proteins. UBB ^+1^ blocks ubiquitin‐dependent proteolysis by interacting with E2‐25k/Hip‐2 and might induce neurotoxicity and the abnormal accumulation of proteins. BAG1 and Hsp70 regulate the proteasomal degradation of tau. UCHL1 accelerates β‐secretase degradation and affects APP processing and Aβ production. USP14 inhibits proteasome activity. Aβ40 and Aβ42 oligomers decrease the trypsin‐like, chymotrypsin‐like and peptidylglutamyl‐like activity of the proteasome and promote intraneuronal accumulation of Aβ conjugates. Low levels of CHIP promote the accumulation of phosphorylated tau species, inhibiting the proteasome and decreasing protein degradation.

Protein misfolding and changes in cellular protein homeostasis are the primary causes of AD. Currently approved medications for treating AD provide symptomatic relief but fail to influence disease progression 63. In the last 10 years, drug discovery has been directed towards developing disease‐modifying agents. In fact, more than 200 medications are currently in phase 2 and 3 clinical trials 64. These compounds can be largely categorized into anti‐amyloid agents and medications that target other pathophysiological pathways (Tables 1, 2, 3, 4, 5, 6, 7). Developments in AD prevention and treatment include anti‐amyloid drugs; methods to hyperphosphorylate tau; and neuroprotective and neurotrophic approaches, such as those involving neurotrophins, neurotrophic factors and stem cells 65. Neurotrophic agents such as cerebrolysin modulate the apoptosis rate of neurons in patients affected by neurodegenerative diseases and are a novel treatment option for AD 66. The accumulation of toxic amyloid is regulated by the quality control systems of the cell (molecular chaperones, autophagy and the UPS) 22. Targeting different components of these pathways will lead to potential therapies for AD (Fig. 5).

**Table 1:** Agents that target Aβ production or aggregation

Agents	Details	Agents under investigation	Status	Ref.
Agents that decrease production of Aβ	β‐secretase inhibitor	MK8931 NIC5‐15	(III)^a^ (II)^a^	72
	γ‐secretase inhibitor	EVP0962 CHF5074	(II) (II)	
	α‐secretase activator	Etazolate EGCg	(IIA) (II/III)^a^	
Agents that decrease aggregation or oligomerization of Aβ	Anti‐aggregation nanopeptide	Tramiprosate	(III)	72, 73
	Fluorinated nanoparticles	–	–	

a The status of a drug in an ongoing clinical trial.

**Table 2:** Other agents that target Aβ metabolism

Targets of the agents	Details	Agents under investigation	Status	Ref.
Apolipoprotein (ApoE) and Aβ clearance	Nuclear receptor modulator and ApoE activator	Bexarotene	–	74
Enzymes that degrade Aβ (NEP, IDE, ECE, ACE)	Increases NEP activity	Valproic acid Oestrogen Green tea	–	75
Aβ transport	Antibodies against LRP	–	–	76
	Intraneuronal Aβ transport	Cerebrolysin	–	77
Aβ signaling	Blocks Fyn kinase or cellular prion proteins	Saracatinib	(II)	76
Intravenous immunoglobulin	Clearance of Aβ	–	–	78
Medications that influence Aβ blood–brain barrier transport	Inhibition of RAGE	–	–	64
	Activation of LRP‐1			

**Table 3:** Immunotherapy‐based agents

Agents	Details	Agents under investigation	Status	Ref.
Active immunotherapy agents (Vaccine)	DNA vaccine	pCMVE/MDC‐3Aβ11 PADRE‐C3d	–	103
	Epitope‐/protein‐/VLP‐based vaccine	Adeno‐10× Aβ3 – 10+ CpG	–	
	Prime‐boost approach	Aβ1‐42 peptide prime/Aβ1‐42 DNA + QuilA	–	
Passive immunotherapy agents	Monoclonal antibody	Bapineuzumab Solanezumab	(III)	64

**Table 4:** Agents that target tau

Targets of the agents	Details	Agents under investigation	Status	Ref.
Hyperphosphorylation	Kinase inhibitor GSK‐3α GSK‐3β CDK5	Lithium AZD‐1080 Minocycline Cerebrolysin	–	64, 104
	Inhibits histone deacetylase	HDAC6	–	73
Microtubule stabilizers	Improves axonal transport, microtubule density	Epothilone NAP (NAPVSIPQ) D‐SAL (SALLRSIPA)	–	76
Blocking tau oligomerization	Reduce tau–tau interactions	Astemizole Lansoprazole Methylene blue	–	
Enhancing tau degradation	Inhibit heat shock protein 90	Curcumin	–	
Vaccination therapy	Promote immunological clearance of tau	–	–	

**Table 5:** Agents that target neurotransmitter signaling

Targeted neurotransmitter		Agents under investigation	Status	Ref.
Acetylcholine		Latrepirdine Huperzine A	(III)	76
GABA		SGS742	(II)*	
		ADS‐8704 (NMDA antagonist)	(III)	
Serotonin	5HT1A	Lecozotan	(II)	
	5‐HT4	PRX‐03140 TD‐8954	–	
	5‐HT6	SB‐742457	(II)	
Histamine		PF03654746 AB288	(I) (II)	
Adenosine		SCH58261	–	

**Table 6:** Agents that target cellular metabolic pathways

Agents	Details	Agents under investigation	Status	Ref.
Agents that target intracellular signaling cascades	Phosphodiesterase inhibitor	AVE‐8112 BCA‐909 THPP‐1	–	76
	Lipoprotein inhibitor	Rilapladib (NCT01428453	(II)	
Agents that target mitochondrial dysfunction	Mitochondrial‐targeted ROS scavenger therapy	Szeto‐schiller peptide (SS‐31)	–	
	Lipoic acid omega‐3 fatty acid combination therapy	NCT01780974 NCT01058941	(I/II)	
Agents that target epigenetics	DNA methylation and histone modification	Vitamin B	–	115
Agents that target oxidative stress	Vitamin E + selenium	NCT00040378	(III)	
	Melatonin	NCT00940589	(II)	
Caspase inhibitors	Neuroprotective benefits	–	–	
Metal chelators	Inhibit zinc and copper from binding to Aβ	Clioquinol	(II)	116
	Metal protein attenuation	PBT2	(II)	

**Table 7:** Agents with other targets

Agents	Details	Agents under investigation	Status	Ref.
Glial cell modulators	Direct glial target	ONO‐2506	–	64
	RAGE receptor antagonist	TTP 488	–	
	TNFα agonist	Enbrel	–	
Anti‐inflammatory agents	NSAID	CHF5074 and SC560	–	
Cholesterol‐lowering agents	Pleiotropic effect	Simvastatin Atorvastatin	(III)	116
Neurogenesis	Pleiotropic effect	Cerebrolysin	–	76
Multi‐target‐directed ligands	Multifunctional compound	Ladostigil (TV3326)	–	
Neurotrophins	NGF brain delivery *via* adeno‐associated virus vector system	CERE‐110 NCT00876863 NCT00087789	(I)^a^	124
	Based on stereotactic implantation containing NGF‐producing cell	NsG0202	(I)	
Nutritional supplements	Combination medical food	Souvenaid^®^	–	125
	Octanoic acid or caprylic acid	Axona^®^	–	
Natural products, *e.g*. quercetin				96, 126
Neuroprotective gonadotropin hormone				76
NOS modulators				
Nucleic acid agents				
Agents that modulate synaptic plasticity and nerve growth				
Stem cells				127

a The status of a drug in an ongoing clinical trial.

### Therapies targeting misfolding

Neurodegenerative diseases share common cellular and molecular mechanisms, such as protein aggregation and inclusion body formation. The protein aggregates consist of fibrils of misfolded amyloid proteins that have taken on a β‐sheet conformation. The formation of these aggregates can be prevented by two pathways: the Hsp40/Hsp60/Hsp70 pathway mediates protein aggregation, while the proteolytic system removes misfolded proteins 67. Several agents may be able to target the protein misfolding pathway. For example, Congo red binds to proteins with β‐sheet structures, alters the protein misfolding pathway and reduces toxicity *in vivo* 24, 68, 69, 70, 71. Recent therapeutic developments include peptides that interfere with the post‐translational modifications that are responsible for the misfolding and aggregation of Aβ and tau. They also include the up‐regulation of molecular chaperones to increase the clearance of protein aggregates 69. Of the ATP‐dependent endogenous proteasome activators (PA700, PA200 and PA28), targeting PA700 may be the most successful therapeutic approach for increasing misfolded protein degradation 79.

### Therapies targeting the different quality control systems of the cell

Ubiquitin‐dependent protein degradation occurs *via* two major catabolic systems: the endosomal/lysosomal system and the ATP‐dependent, non‐lysosomal proteolysis system, which is known as the UPS.

#### Autophagy (the endosomal/lysosomal system)

Through autophagy, lysosomes degrade normal and aggregated proteins that are usually found under conditions of stress or injury. Independent alterations in the endocytic pathway activate the lysosomal system and increase the number and density of lysosomes as well as gene expression 80. The latter effect results in the synthesis of all classes of lysosomal hydrolases, including cathepsin 81. Lysosomal cathepsin B is up‐regulated either by the accumulation of proteins or by the modulator 2‐Phe‐Ala‐diazomethyl ketone (PADK). Systemic administration of PADK increases cathepsin B activity, which increases the clearance of intracellular Aβ and decreases its extracellular deposition. PADK inhibits protein accumulation and promotes synaptic integrity *in vivo* and *in vitro* 82. Thus, modulators of lysosomal activity show great promise for the treatment of neurodegenerative diseases.

Autophagy reduces the accumulation and facilitates the clearance of normal or mutant Aβ 80. The Aβ that is generated in endosomes and autophagic vacuoles is delivered to lysosomes, where it is cleared through lysosomal proteolysis under normal conditions 83. Enhancing lysosomal proteolysis in TgCRND8 transgenic mice increased the clearance of autophagy substrates, which reduced intracellular and extracellular Aβ levels and reversed multiple cognitive deficits 84. Rapamycin could be effective in preventing or reversing AD pathology, because it inhibits motor activity and enhances autophagy. Rapamycin also inhibits NFT formation and tau phosphorylation and reduces cognitive impairment 80. Hence, drugs that induce autophagy could reduce or eliminate protein aggregates 85. Impaired clearance and accumulation of autophagic vacuoles in AD‐affected neurons causes abnormal autophagy 86, 87; this effect might block the neuroprotective effect of autophagy and limit the accumulation of toxic proteins (including tau), thus promoting neuronal cell death 88.

#### Molecular chaperones (Hsps)

Molecular chaperones and the UPS are the first and second lines of defense against protein misfolding and aggregation. Chaperones regulate the folding of newly synthesized proteins and the refolding or transfer of misfolded proteins to protein degradation systems 22. Lower molecular weight Hsps (12–43 kD) are ATP independent, whereas higher molecular weight Hsps (>43 kD) are ATP dependent. Several studies 89, 90, 91, 92, 93 have demonstrated that the chaperone network can be targeted to develop therapeutic strategies for AD 94. Chaperones bind to Aβ and tau and regulate their degradation. Both Hsp90 and Hsp70 participate in APP metabolism 95.

Hsp70 is ATP dependent and the major target for AD treatment. Increased Hsp70 levels inhibit its ATPase activity and might be an effective treatment strategy for AD 94. In 5XFAD transgenic mice, recombinant human Hsp70 reduces Aβ plaque formation 96. At very high concentrations, methylene blue inhibits Hsp70 and increases tau clearance 68. Curcumin was also shown to increase Hsp70 and Hsp90 activity, which could inhibit or delay amyloid formation and reduce neuronal death 97, 98. The co‐chaperone protein Bcl2‐associated athanogene (BAG‐1) forms a complex with Hsp70 and tau and could inhibit tau degradation 99 in cell cultures, increasing both tau and APP levels 99, 100.

Hsp90 regulates the misfolding and stabilization of neurotoxic proteins and facilitates tau pathology in AD 101. Inhibiting Hsp90 reduces levels of hyperphosphorylated and Ser/Thr‐mutated tau as well as of the kinases that are involved in persistent hyperphosphorylation 102. Moreover, Hsp90 inhibition induces heat shock factor, Hsp70 and small heat shock proteins (sHsps). This inhibition also facilitates Hsp70 binding to abnormal proteins to form a complex that is ubiquitinated by CHIP (carboxyl C‐terminus of Hsp70‐interacting protein) and degraded through proteolysis. Thus, Hsp90 inhibition increases tau degradation and might be a potential therapeutic target for tau‐based neurodegeneration in AD 101. The antibiotic geldanamycin was the first Hsp90 inhibitor to be identified; geldanamycin was shown to compete with ATP and stop neurotoxic protein folding and stabilization 86, 87, 105, 106. Although geldanamycin is toxic and cannot penetrate the blood–brain barrier, other agents have been developed. 17‐AAG and PU‐DZ8 are synthetic purine scaffold proteins that cross the blood–brain barrier, inhibit Hsp90, and decrease levels of phosphorylated tau 107.

#### The ubiquitin proteasomal system

The role of the UPS in AD has been demonstrated through observations of the downregulation of E1 and E2 enzymes, the oxidation of DUBs, the accumulation of mutated ubiquitin, the detection of proteasome subunits and decreased proteasomal activity 43. Toxic Aβ is formed when β‐secretase cleaves APP and generates a C‐terminal fragment (APPCTFB or C99). This fragment is subsequently cleaved by γ‐secretase to produce toxic Aβ40 and Aβ42. Proteasome inhibitors up‐regulated APP C99, suggesting that they may decrease β‐secretase activity 62.

### UPS components as potential therapeutic targets in AD management

In the pathogenesis of AD, it remains unclear whether Aβ accumulation causes UPS dysfunction or vice versa 13, 92.

The 26S proteasome ubiquitinates and degrades proteins other than β‐ and γ‐secretase that are involved in APP metabolism 20. The overexpression of an APP mutant isoform has been correlated with increased oxidative stress and decreased proteasome activity 84. Moreover, proteins associated with AD (most notably AHNAK, gelsolin and tenascin‐R) were found to exhibit strong interactions with ubiquitin C. This change in AD associated proteins was observed in regions that are frequently affected in AD (the hippocampus, parietal cortex and cerebellum), suggesting that ubiquitin C promotes age‐related neurodegeneration 108. Aβ40 and Aβ42 oligomers, but not their monomers, also inhibit proteasomal activity in cell‐free assays 22.

The UPS processes and degrades presenilin‐1 (ps‐1), presenilin‐2 (ps‐2) and mutated presenilin, which are key proteins in the pathogenesis of AD. Therefore, proteasomes and mutated presenilin might indirectly influence Aβ production 30. Ubiquilin regulates UPS activity and presenilin metabolism. It decreases γ‐secretase activity and downregulates presenilin and its N‐terminal and C‐terminal fragments 13. Ubiquilin‐1 is functionally associated with AD and interacts with both ps‐1 and ps‐2 13.

The UPS ubiquitinates and degrades β‐secretase 109. Up‐regulating proteasomal degradation of β‐secretase and γ‐secretase decreases Aβ levels. Proteasome activation can also be used to clear tau 85. Hence, the UPS may provide an alternative target for new therapies for AD 13. An age‐dependent decrease in proteasomal activity may cause both Aβ and tau to accumulate. Once Aβ and tau aggregates are formed, they decrease proteasome activity and create a vicious cycle that increases Aβ and tau accumulation 110. Normal tau in the normal brain and hyperphosphorylated and misfolded tau in the AD brain are present at both pre‐ and postsynaptic terminals 111. Abnormally folded and hyperphosphorylated tau accumulates in dendrites, axons and somas 112. In addition, changes in proteasomal subunit composition alter proteasomal activity in AD 33. Thus, tau oligomers might accumulate in AD synapses because of increased proteasomal components and UPS dysfunction 111. BAG‐1 and Hsp70 regulate the proteasomal degradation of tau 22. Kinases (such as glycogen synthase kinase‐3 and casein kinase‐1) phosphorylate tau 26 and might be candidate therapeutic targets for AD. Tau co‐immunoprecipitates with CHIP, an E3 ubiquitin ligase that ubiquitinates tau before the proteasome degrades it. Phosphorylated tau accumulates in the neurons of CHIP knockout mice and inhibits the interaction between tau and CHIP in AD 113. *In vitro* studies of AD brains showed that high CHIP levels were associated with less tau aggregation and fewer NFTs 114. In addition, the *CHIP* knockout mice showed the accumulation of species of phosphorylated tau 113. Thus, the proteasome is directly involved in NFT accumulation, and reduced proteasomal activity might cause tangle formation in patients with AD 43.

Ubiquitin C‐terminal hydrolase 1 is oxidized and downregulated in AD 110, and UCHL1 inhibition up‐regulates β‐secretase. UCHL1 also accelerates β‐secretase degradation, impairs APP processing and decreases Aβ 109. Furthermore, the synaptic dysfunction that is induced by Aβ can be reduced by increasing UCHL1 expression 110. Hence, UCHL1 is a promising target for the development of therapeutic agents for AD.

### Importance of UPP in the management of Alzheimer's disease

Amyloid beta neurotoxicity inhibits the proteasome in association with increased brain levels of E2‐25k/Hip‐2. E2‐25k/Hip‐2‐mediated proteasome inhibition can directly induce neuronal death and indirectly increase Aβ production 45. *In vitro*, E2‐25k/Hip‐2‐mediated ubiquitination of a mutated form of ubiquitin (UBB^+1^) at Lys48 was suggested to inhibit proteasome activity 87. *In vitro*, cultures exposed to Aβ42 up‐regulate E2‐25K/Hip‐2 expression in neurons 117. E2‐25k/Hip‐2 induction is required for neuronal cell death under Aβ toxicity. It inhibits proteasome activity and promotes the accumulation of ubiquitin conjugates, which increases the levels of apoptosis signal regulating kinase‐1/C‐Jun N‐terminal kinase and leads to Aβ neurotoxicity. Therefore, E2‐25k/Hip‐2 might be a potential therapeutic target in AD 118.

Ubiquitin is an endogenous reporter of proteasome activity in human AD pathology and can inhibit proteasomal degradation 43. Current studies have shown that UBB^+1^ accumulates in the brains of AD patients 43. UBB^+1^ is selectively expressed in neurons and competes with normal ubiquitin for binding to proteasomal target proteins. It also interacts with E2‐25k/Hip‐2 to inhibit the 26S proteasome and block ubiquitin‐dependent proteasomal proteolysis 61, 119. Polyubiquitin chains that contain UBB^+1^ are refractory to DUB lysis and alter proteasomal degradation of the substrate 120. UBB^+1^ also impairs mitochondrial movement and promotes neurodegeneration in primary neurons 121. Proteasome‐associated proteins contribute to the proteasome's proteolytic function and regulate its activity through calcium signaling. The function of these proteasome‐associated proteins was also impaired in AD brains 30.

The loss of proteasome activity with age leads to decreased subunit expression, alterations and replacement of proteasome subunits and the formation of inhibitory cross‐linked proteins 122. In transgenic (Tg2576) mice, ubiquitin and proteasome activity decreased with age, whereas Aβ42 levels increased. In addition, in cultured neurons, extracellular administration of Aβ markedly decreased proteasome activity 123.

### Recent therapeutic developments

Enhancing chaperone function, chaperone up‐regulation and aggregate clearance or targeting post‐translational modifications (inhibiting oxidation and phosphorylation) could inhibit protein misfolding and aggregation. For example, current AD therapeutic strategies that target Hsp70 include the induction of endogenous Hsp70, exogenous Hsp70 utilization and Hsp70 inhibition. The combination of agents that affect more than one of these therapeutic targets will be the most effective treatment strategy for AD 69.

E3 enzymes determine the specificity of UPS‐mediated proteolysis. Hence, identifying their specific allosteric modulators could provide an effective therapeutic target 21, 69. PROTACS (proteolysis‐targeting chimeric molecules) bind to E3 ubiquitin ligases. They promote the synthesis and attachment of the poly‐ubiquitin chain to the target protein, which is followed by protein degradation by the 26S proteasome. Therefore, developing PROTACS could prevent the accumulation and aggregation of tau proteins 128.

Resveratrol is a phytoalexin polyphenolic compound (trans‐3, 4, 5‐trihydroxystilbene) from grapes and wines. As a result of its anti‐amyloidogenic activity, resveratrol might show therapeutic potential in AD 129. Resveratrol promotes neuroprotection *via* multiple mechanisms 13: it inhibits Aβ aggregation, scavenges oxidants, reduces inflammation 13 and removes Aβ *via* the proteasomal pathway 129.

Betulinic acid, a lupine‐type pentacyclic triterpene derived from birch trees, activates the chymotrypsin‐like activity of the proteasome 130. Overexpressing arsenite‐inducible RNA‐associated protein, which stabilizes proteasome activity in the absence of ATP 131, is another therapeutic approach to maintain proteasome function.

## Future prospects

Emerging evidence suggests that the substrate‐specific components of the UPS, such as the E3 ligase, may impair ubiquitination, which results in UPS aberrations, defective proteasome‐mediated protein degradation, the accumulation of toxic proteins and neuronal death. Studies of the interaction between Aβ and the proteasomal system have shown that Aβ40 binds directly to the inside of the 20S proteasome, blocks the peptide channel and inhibits the chymotrypsin‐like activity of proteasome 132. Tau accumulation also inhibits the proteasome and reduces protein degradation 18, 133. Several proteases break down tau, including calpain; caspases 3, 6 and 9; cathepsin D and L; and puromycin‐sensitive aminopeptidase. These proteases limit tau proteolysis and may contribute to AD pathology. However, they may also be potential therapeutic targets for this disease 134.

The development of proteasome activators is another potential approach to prevent Aβ accumulation in AD 135. Proteasome‐activating peptide 1 increases the chymotrypsin‐like activity of the proteasome in fibroblast cell cultures. It also down‐regulates and prevents the aggregation of oxidized proteins in amyotrophic lateral sclerosis 135.

USP14, a proteasome‐associated DUB, inhibits the degradation of ubiquitin‐protein conjugates both *in vivo* and *in vitro*. USP14 inhibits proteasome activity by trimming the ubiquitin chain on the substrate. Inhibition of USP14 accelerates the degradation of oxidized proteins and increases cell resistance to oxidative stress 136. IU1 is a chain‐trimming enzyme that inhibits USP14 and prevents the rescue of ubiquitinated forms of the neurotoxic proteins tau and ataxin. IU1 acts on the proteasome and was found to have a therapeutic benefit in AD 14. IU1 was also shown to promote the degradation of several overexpressed proteins (tau, TDP‐43 and ATXN3) in various neurodegenerative diseases 27. As ubiquitin chain trimming can promote or inhibit proteasomal degradation, future studies should identify whether IU1 inhibits other DUBs or proteases in human cells 27.

## Conclusion

The UPS and its components are key factors in AD treatment. The UPS mediates the pathogenesis of AD through various mechanisms. Full understanding of the mechanisms of proteasomal activity is critical for the development of improved therapeutic and diagnostic strategies for managing AD. The development of effective medications also requires comprehensive understanding of the role of proteasome inhibition and how neuronal death occurs in patients with AD. Amyloid beta has been shown to inhibit proteasomes *in vitro*, even though it remains unclear whether AD patients show the same pattern. Future AD therapies should reduce protein aggregation by targeting specific components of the UPS (*e.g*. the 26S proteasome, ubiquitin and DUBs). Another strategy could be to enhance proteasome activity by inhibiting USP14. To develop new therapies for AD, we need to fully understand how the components of the UPS interact in protein degradation.

## Conflicts of interest

All of the authors declare that they have no potential conflicts of interest to disclose.
